# The Relationship between Environmental Regulation, Green-Technology Innovation and Green Total-Factor Productivity—Evidence from 279 Cities in China

**DOI:** 10.3390/ijerph192316290

**Published:** 2022-12-05

**Authors:** Yuhua Ma, Tong Lin, Qifang Xiao

**Affiliations:** College of Economics, Sichuan Agricultural University, Chengdu 611130, China

**Keywords:** dual environmental-regulation, green technological innovation, green total-factor productivity, spatial spillover effect, threshold effect

## Abstract

This paper employs the SBM-DDF method to measure the index of green total-factor productivity (GTFP), based on the panel data of 279 prefecture-level cities in China from 2007 to 2019, and constructs a spatial Durbin model (SDM) and a threshold effect to empirically test the effects of dual environmental-regulations and green technological innovation on GTFP. The results are as follows: (1) the SDM supports a nonlinear contribution of dual environmental-regulations spillover to GTFP. The relationship between formal environmental-regulation and GTFP is an inverted U-shape, while a U-shaped nonlinear relationship is found between informal environmental regulation and GTFP. (2) Green technology innovation has a significant negative moderating effect on the process of dual environmental-regulations affecting GTFP in local regions, but a positive moderating effect on informal environmental regulation in neighboring regions. (3) There is a significant green technology innovation threshold effect of dual environmental-regulations affecting GTFP. Specifically, the promotion effect of dual environmental-regulations on GFFP gradually increases as the level of green technology innovation increases.

## 1. Introduction

Since the reform and opening up, the development of China’s economy and society has made great achievements. However, while the economy continues to grow at a high rate, problems such as environmental pollution, waste of resources, and ecological imbalance have emerged, and the environmental carrying-capacity between regions is overwhelmed. China’s economic development faces enormous challenges. The economic mode of “high consumption, high pollution, and high emissions” at the expense of resources, the environment, and people’s well-being, is no longer appropriate for the current stage of China’s development. In order to achieve green development, China needs to change the development mode to break the bottleneck of green development and achieve high-quality economic development. The 17th Party Congress formally put forward the new requirement of “building an ecological civilization” for the first time; the 18th Party Congress incorporated “ecological civilization” into the overall layout of the five-in-one; the 19th Party Congress further brought the construction of ecological civilization to a new height. Moreover, the “14th Five-Year Plan” further pointed out the goal of achieving new progress in the construction of ecological civilization and green development. Therefore, the green total-factor productivity, which considers both environmental and economic benefits, is the key to green development, and the essential path to achieving a win-win situation for both energy saving and economic growth [1]. With “green water and mountains”, there will be “golden mountains and silver mountains”. Under the tightening of resources and environment, how to further improve green total-factor productivity, win the battle against pollution, and promote green and sustainable economic development is an enormous challenge for China and a crucial problem that needs to be solved.

Due to the negative externalities of environmental pollution and the failure of market regulation, environmental regulation has come into being. As an effective way to solve the negative externality of environmental pollution [2], environmental regulation is an important policy tool for the government and the public, to maximize social welfare and plays a significant role in pollution reduction. Gollop and Robert, Gary and Shadbegian directly equated environmental regulation with formal environmental regulation under government domination [3,4]. Pargal and Wheeler proposed the concept of informal environmental regulation [5]. In addition, New Institutional Economics regards the institution as a social-game rule composed of formal rules and informal rules [6]. Accordingly, environmental regulations can be divided into formal and informal environmental-regulations. However, the government is forcing environmental regulations while also paying for them. This is mainly represented in the following aspects: first, the government needs to intervene in environmental issues for the sustainable development of the economy. The government directly controls the behavior of micro-market economic agents. The strict environmental regulations will not only reduce the level of environmental pollution, but also promote the development of environmental protection and introduce advanced equipment to improve the competitiveness of enterprises [7]. Second, since the environment is a classical public-good, the government should play a leading role in environmental regulation and increase its investment in public goods and public services in the environmental field [8]. Third, the public’s general participation is an essential guarantee for the realization of environmental regulation. When implementing environmental regulations, the government should also raise public awareness of environmental protection, increase environmental education and publicity, and develop a complete environmental-regulation mechanism. Based on the above, the environmental regulation involved in this paper mainly consists of two parts: formal environmental regulation and informal environmental regulation. On the one hand, in order to protect and improve the environment, China has introduced formal-environmental-regulation policies such as the Environmental Protection Law and the Air Pollution Prevention and Control Law, in an attempt to win the battle against pollution. On the other hand, the problem of environmental pollution is also closely related to the life of the public. With the increase in public awareness of environmental protection and the increase in channels to participate in environmental protection, informal environmental regulation on behalf of the public is springing up and gradually highlighting its role in environmental protection. Therefore, will the implementation of dual environmental-regulations promote or hinder the growth of green total-factor productivity? Porter and Vanderlinde proposed the famous Porter hypothesis [9]. In the subsequent study of the Porter hypothesis, Jaffe and Palmer first distinguished among the “weak,” “narrow,” and “strong” versions of the Porter hypothesis [10]. The “narrow” version of the Porter hypothesis states that flexible regulatory policies will give enterprises more incentives to innovate than traditional forms of regulation. The “weak” version of the Porter hypothesis” states that environmental regulation may induce firms to innovate, but such innovation does not necessarily increase firm productivity. The “strong” version of the Porter hypothesis states that environmental regulation can enhance the competitiveness of firms through technological innovation, and increase their total-factor productivity. Based on this, does the level of green innovation have a moderating effect on environmental regulation and green total-factor productivity? Research on the above issues can help uncover the institutional factors for the validity of the Porter hypothesis and help explore an effective path to enhance green total-factor productivity.

## 2. Literature Review

The current research on environmental regulation, green-technology innovation, and green total-factor productivity includes the following three main aspects. Firstly, there are the studies on environmental regulation and green total-factor productivity. The existing studies have presented three main perspectives. The first is the “compensation hypothesis”, which states that environmental regulation will promote green total-factor productivity. Cheng et al. examined the impact of environmental regulations on GTFP from a spatial perspective, by dividing them into command-based and market-based environmental regulations, showing that both boost the expansion of GTFP and that the combination of command-based and market-based environmental regulations is more favorable to GTFP [11]. Lee, C. et al., Lena et al., and Tong et al. all pointed out that environmental regulation made a significant contribution to GTFP [12,13,14]. The second is the “green-paradox hypothesis”, which states that environmental regulations will hinder the improvement of green total-factor productivity. Rexhauser and Rammer revealed that environmental regulation could even cause a decline in productivity due to increases in production costs [15]. Yuan et al. pointed out that environmental regulation reduced patent output and R&D investment, and hindered technological innovation. The “weak” version of the Porter hypothesis is not supported. In the long-term, environmental regulation improved energy efficiency but hindered labor productivity. The “strong” version of the Porter hypothesis is not supported [16]. Zhan et al. proposed that mandatory environmental regulation had a significant negative effect on the improvement of green-development efficiency by changing technical progress [17]. Lanoie et al. provided an empirical analysis, and found that environmental regulation on productivity was negative, while the opposite result was observed with lagged regulatory-variables in Quebec [18]. The third is the non-linear hypothesis, which states that environmental regulation has asymmetric effects on green total-factor productivity. A large number of scholars have argued that environmental regulations have a U-shaped effect on green total-factor productivity [19,20,21,22] or an inverted U-shaped effect [23,24,25,26,27]. However, Brännlund found environmental regulation had no significant impact on the productivity of manufacturing enterprises in Sweden [28]. Secondly, there is the research into green-technology innovation and green total-factor productivity. With the development of the economy and society, problems such as energy consumption and pollution are becoming more serious. Many scholars have found that technological innovation can break technological barriers and blockades, and green-technology innovation has brought new opportunities for green economic development. With data from Chinese A-share businesses, Wu et al. showed that green-technology innovation boosted green total-factor productivity, but that there was a threshold effect-of-technology gap, and that green-technology innovation inhibited green total factor-productivity below the threshold [29]. Wang et al., Song et al., and Jiakui et al. all concluded through empirical research that technological innovation significantly increased green total-factor productivity [30,31,32]. Santra found innovation technology had a sound impact on the sustainable performance of BRICS countries and that green technological innovation helped firms and countries to reduce their energy absorption and CO_2_ emissions [33]. Meirun et al. reported that green-technology innovation in Singapore had a positive and significant impact on economic growth, and a negative effect on carbon emissions [34]. Suki et al. also found green-innovation technology minimized environment-degradation, optimized resource-utilization, and improved the overall productivity in ASEAN-6 countries [35]. Further, Xiao et al. used the spatial Durbin model and threshold model to test, and discovered that there was a dynamic effect of technological progress on agricultural green production, with an inverted U-shaped relationship for green production in the local region, but a U-shaped relationship in the neighboring region [36]. Thirdly, there are the studies on environmental regulation and green-technology innovation. Some scholars contended that environmental regulation forced enterprises to engage in technological innovation while increasing costs, and this had a significant direct-positive-effect on technological innovation [37,38,39,40]. However, the traditional neoclassical theory argued that environmental regulation increased the costs of enterprises, leading to difficulties in capital turnover, resulting in lower profit levels, and having a detrimental impact on technological innovation [41,42]. Other scholars believed that environmental regulation had a non-linear effect on the level of innovation [43,44,45]. Yi M et al. argued that environmental regulation of various intensities had a threshold effect on green-technology innovation, and it was possible to promote green-technology innovation when environmental regulation remained within a reasonable range [46].

A review of the above literature reveals that scholars have conducted richer studies on environmental regulation, green technological innovation, and green total-factor productivity, and have made different interpretations of the relationship between the three. However, scholars still disagree widely on the effects of environmental regulation, green-technology innovation, and green total-factor productivity. Most scholars have only explored the relationship between the first two, and relatively few studies have integrated the three into a unified framework. Based on the shortcomings of the above studies, this paper will try to promote relevant research from the following aspects: (1) Constructing the index system of green total-factor productivity, and using SBM-DDF to measure the green total-factor productivity index of 279 prefecture-level cities in China from 2007 to 2019. (2) From a spatial perspective, dividing environmental regulation into formal and informal environmental-regulations, and examining the spatial-spillover effect and heterogeneity of dual environmental-regulations on green total-factor productivity from theoretical and empirical dimensions. (3) Introducing green technological innovation as a moderating- and threshold-variable, to explore its moderating- and threshold-effects between dual environmental-regulations and green total-factor productivity.

## 3. Research Hypothesis

### 3.1. Formal Environmental Regulation and Green Total-Factor Productivity

Formal environmental regulation means that laws or norms concerning environmental protection are made with government departments as the leading authority and constrained by public power, reflecting the government’s initiative to protect the environment. Specifically, it includes emission standards for wastewater and waste gas, environmental audits, environmental surveillance, and pollution-tax collection, etc. [47]. The relationship between air pollution and real income is hypothesized in the environmental Kuznets curve (EKC), which was first introduced by Grossman and Krueger [48]. The EKC hypothesis suggests that economic growth mainly affects environmental pollution through scale, structural and technological effects. Environmental quality and economic development show an inverted-U-shaped relationship. This means that pollution increases in the initial stages of economic growth, and then begins to decline when economic growth reaches a certain level. Therefore, there may also be an inverted-U-shaped relationship between the effect of environmental regulations and green total-factor productivity, from the EKC theory. The impact of environmental regulation on GTFP is mainly through two mechanisms: the “innovation compensation” effect and the “compliance cost” effect. The “innovation compensation” effect is based on the Porter hypothesis, which holds that a reasonable environmental-regulation policy will increase the incentive of enterprises to innovate, enhance their technological innovation, and then improve their productivity, offset the increased costs, and increase their profitability. The “cost-of-compliance” effect is based on neoclassical economic theory, which suggests that environmental regulation will drive up the cost of environmental protection, thus crowding out firms’ investment in innovation and R&D, resulting in lower productivity and reduced profit-margins. Specifically, in the initial stages of formal environmental regulation, strict environmental regulation will increase the pressure and incentive for companies to innovate and achieve technological progress. The improvement of the enterprise technology innovation-level will produce technological effects, specifically in two aspects: First, it will improve the efficiency of resource use, reduce the factor input per unit of output, and weaken the impact of production on nature and the environment; Second, the continuous development of clean enterprise-technology and the effective recycling of resources will reduce the pollution emissions per unit of output. Technological effects will force enterprises to focus more on energy conservation and emission reduction. At this moment, the “innovation compensation” effect exceeds the “compliance cost” effect. From the perspective of the enterprises’ own development, their technological progress has brought an increase in production, a decrease in total-energy consumption, and an increase in the level of green total-factor productivity. However, with the progressive rise in the stringency of environmental regulations, the growth of the green economy will require a large amount of innovative investment, which will result in scale effects. The cost of environmental protection and the investment of resources for enterprises will rise, and enterprises may crowd out their funds for innovation and green innovation to cut costs and maintain their original profit-levels. At this time, the “innovation compensation” effect lags behind the “compliance cost” effect. In order to meet environmental standards, enterprises will consider terminal ways to reduce emissions, leading to a lack of incentive to innovate, which is not conducive to the improvement of green total-factor productivity levels. Based on the above analysis, this paper puts forward the following hypothesis:

**Hypothesis** **1** **(H1).** *There is an inverted-U-shaped relationship between formal environmental regulation and green total-factor productivity.*

### 3.2. Informal Environmental Regulation and Green Total-Factor Productivity

Informal environmental regulation is a restraint and a monitoring mechanism formed by the public, media, environmental organizations, etc., for environmental protection, including public complaints, public pressure, and the boycott of polluting enterprises’ products [49], which was first proposed by Pargal and Wheeler [5]. Informal environmental regulation is an important force for the external supervision of polluting enterprises, reflecting the public’s consciousness of protecting the environment, and effectively complementing the inadequacy of formal environmental regulation. Informal environmental regulation affects green total-factor productivity through two main pathways. The first is direct impact, which means that through public pressure and media exposure, enterprises are prompted to change their production methods, reduce the emission of pollutants and improve environmental quality. The second is indirect impact, which means that the public reports enterprises whose emission standards do not meet the requirements to the environmental-protection authorities, so as to prompt polluting enterprises to meet the standards by legal means, and realize the green production of enterprises. However, in the early stage, the public’s environmental awareness and environmental monitoring are still in the nascent stage; the environmental-monitoring efforts are only “immediate”, and can’t effectively reflect the pollution behavior of enterprises. In addition, some public or group organizations lack holistic awareness, and tend to deal with issues based on personal interests or group interests without considering the interests of society. Public appeals to companies for compensation costs in excess of the legal requirements will result in a rapid rise in costs for companies in the short-term. Moreover, the use of improper and unreasonable ways to expose the environmental behavior of enterprises may also put them under enormous pressure to survive. Therefore, excessive pressure from the public and excessive media exposure will not only seriously damage the image of enterprises, but may also lead to some smaller enterprises being unable to raise funds, facing the risk of closing down or going bankrupt, affecting the development prospects of enterprises, and bringing the improvement of green total-factor productivity to a standstill. With the continuous improvement of the channels for public participation in environmental protection and the support of China’s vigorous promotion of the green concept, public supervision of enterprises’ environmental protection appears to be more active. As the public’s awareness of the rule of law is starting to increase, social organizations will exert influence on enterprises’ energy consumption and emission standards in a more flexible way, prompting enterprises to reduce pollution emissions, which plays an important role in enterprises’ green total-actor productivity improvement. Therefore, this paper puts forward the following hypothesis:

**Hypothesis** **2** **(H2).** *There is a U-shaped relationship between informal environmental regulation and green total-factor productivity.*

### 3.3. Mechanism of Spatial Action

Due to the differences in resource endowment and the unbalanced economic development among regions in China, the intensity of environmental regulations varies across regions, and there is significant regional heterogeneity in green production. On the one hand, according to the pollution haven hypothesis, when the intensity of environmental regulation in local areas increases, polluting enterprises will move to neighboring areas with lower environmental regulations for their development. As a result, environmental pollution and market competition in the neighboring areas will intensify, leading to stagnation of green-technology innovation and a low level of green total-factor productivity. On the other hand, due to the existence of competition for the promotion of officials, the evaluation criteria for the promotion of officials will take into account the economic- and environmental-development performance. The competition model among governments is mainly a regulatory competition model. When local regions increase the intensity of environmental regulation, neighboring regions will follow in their footsteps and change the intensity of environmental regulation accordingly. It will create a model learning-effect for the neighboring regions. In addition, technological innovation resulting from the strengthening of environmental regulation can also have an impact on neighboring regions, meaning that there is a “free-rider” effect [50]. Therefore, environmental regulation may have a facilitating or inhibiting effect on green total-factor productivity in neighboring regions, through spatial-spillover effects. Moreover, environmental pollution tends to spread to neighboring regions, and environmental management similarly has regional externalities. Therefore, the conclusion obtained by introducing spatial factors into the econometric model is more relevant to the reality. Accordingly, this paper puts forward the following hypothesis:

**Hypothesis** **3** **(H3).** *Dual environmental-regulation will affect green total-factor productivity, through spatial-spillover effects.*

### 3.4. Moderating and Threshold Effects of Green Technology Innovation

The R&D- and innovation-capabilities of companies appear to be essential in the impact of environmental regulation on green total-factor productivity. On the one hand, the improvement of green total-factor productivity requires advanced environmental-protection technology and production facilities, while the level of green innovation represents the innovation ability of enterprises. The higher the level of green innovation, the more scientific-research achievements in environmental management, and the greater the technical-support role for green total-factor productivity [51]. Against the background of tightening environmental-regulation policies, the green production-efficiency of enterprises will be constrained by the level of technological innovation. When the level of green technological-innovation of enterprises is improved, enterprises have sufficient conditions to reduce pollution and emissions, and in the process of environmental-regulation policy tools to control pollution emissions, they can obtain a first-mover advantage and differentiation advantage, reduce the pressure of environmental management, and effectively solve the problem of insufficient resources and the high cost of enterprises. On the other hand, enterprises with a high level of green-technology innovation will also create a positive image for enterprises, and send positive signals to the public, as well as to cooperative investors. This is so that enterprises can obtain more resources and revenue, have enough funds to carry out emission-reduction activities under the supervision of informal-environmental-regulatory forces, improve their resource utilization, and achieve optimal allocation. However, it is necessary to note that the level of green innovation requires continuous and high investments in the innovation process, which may increase the financial risk of enterprises and cause the breakage of their financial chains. Green-technology innovation will act as a moderating variable to influence the effect of environmental regulation on green total-factor productivity.

Since the level of green technological-innovation varies greatly across enterprises, the effect of dual environmental-regulations on green total-factor productivity may also have a threshold effect of green technological-innovation. When the level of green-technology innovation is low, the productivity of enterprises is low. At this moment, cleaner production in response to environmental regulations will increase the cost of the enterprise, crowd out the funds for other activities, and hinder the improvement of green total-factor productivity. When the level of green-technology innovation is high, the improvement of enterprise technology will lead to the improvement of enterprise efficiency. Accordingly, it will offset the cost of environmental protection in environmental regulation to a certain extent, and even bring excess profit, which has a positive effect on the improvement of green total-factor productivity. Based on the above analysis, this paper puts forward the following hypothesis:

**Hypothesis** **4** **(H4).** *Green-technology innovation has a moderating role in the process of dual environmental-regulation affecting green total-factor productivity, and green-technology innovation may be a threshold variable for dual environmental-regulation affecting green total-factor productivity.*

**Figure 1:**
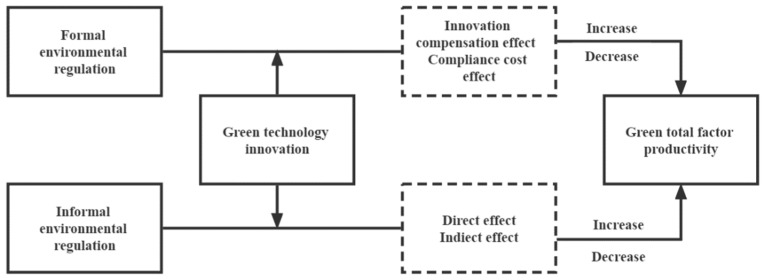
Theoretical framework.

The theoretical framework developed in this paper is shown in Figure 1.

## 4. Method and Design

### 4.1. Model Construction

Based on the above research hypothesis, this paper selects a spatial-econometric model to test the impact of dual environmental-regulation on green total-factor productivity. The spatial-econometric model includes SAR, SEM, and SDM, and the specific model used will be explained by further tests later. The constructed spatial-econometric models are as follows:

(1) $${\mathrm{GTFP}}_{it}\;=\;\rho \sum_{j\;=\;1,j\;\neq \;i}^{n}W_{ij}{\mathrm{GTFP}}_{it}\;+\;\beta_{1}{\mathrm{FER}}_{it}\;+\;\beta_{2}{{\mathrm{FER}}^{2}}_{it}\;+\;\gamma {\mathrm{Control}}_{it}\;+\;\theta \sum_{j\;=\;1,j\;\neq \;i}^{n}W_{ij}({\mathrm{FER}}_{it}\;+\;{{\mathrm{FER}}^{2}}_{it}\;+\;{\mathrm{Control}}_{it})\;+\;\mu_{i}\;+\;\eta_{t}\;+\;\varepsilon_{it}$$

(2) $${\mathrm{GTFP}}_{it}\;=\;\rho \sum_{j\;=\;1,j\;\neq \;i}^{n}W_{ij}{\mathrm{GTFP}}_{it}\;+\;\beta_{3}{\mathrm{IER}}_{it}\;+\;\beta_{4}{{\mathrm{IER}}^{2}}_{it}\;+\;\gamma {\mathrm{Control}}_{it}\;+\;\theta \sum_{j\;=\;1,j\;\neq \;i}^{n}W_{ij}({\mathrm{IER}}_{it}\;+\;{{\mathrm{IER}}^{2}}_{it}\;+\;{\mathrm{Control}}_{it})\;+\;\mu_{i}\;+\;\eta_{t}\;+\;\varepsilon_{it}$$

where Equations (1) and (2) are used to test hypotheses H1, H2, and H3. GTFP denotes green total-factor productivity, FER and IER denote formal environmental regulation and informal environmental regulation, respectively, and Control denotes control variables. *i* and *t* denote region and year, *μ_i_*, *η_t_* denote spatial and temporal fixed-effects, *ε_it_* represents the random-error term, and *β*_1_, *β*_2_, *β*_3_, *β*_4_, *γ* are parameters to be estimated. *W_ij_* is the inverse-distance spatial-weight matrix, p denotes the spatial-autocorrelation coefficient, and *θ* is the spatial-lag-term coefficient. If *p* ≠ 0 and *θ* = 0, the model can be reduced to the SAR model; if *p* = 0 and *θ* = 0, it can be simplified to the SEM model.

### 4.2. Variable Description

#### 4.2.1. Explained Variable

Regarding the measurement of green total-factor productivity (GTFP), this paper draws on the research results of Rusiawan et al. and Ren et al. [52,53] to construct an indicator system for GTFP, and uses the directional distance function SBM-DDF to measure GTFP, based on the studies of Chung et al. and Fukuyama et al. [54,55].

Assume that each measurement unit uses *x* to denote *N* inputs, $x\;=\;(x_{1},x_{2}\ldots x_{n})\in R_{N}^{+}$, *y* to denote *M* desired outputs, $y\;=\;(y_{1},y_{2}\ldots y_{m})\in R_{M}^{+}$, and *z* to denote *K* undesired outputs, $Z\;=\;(z_{1},z_{2}\ldots z_{k})\in R_{K}^{+}$. The SBM-DDF model is constructed as follows:

(3) $$\begin{array}{c} S_{v}^{t}(x_{i}^{t},y_{i}^{t},z_{i}^{t};g^{x},g^{y},g^{z})\;=\;\frac{1}{3}\underset{s^{x},s^{y},s^{z}}{\max}\left[\frac{1}{N}\sum_{n\;=\;1}^{N}\frac{s_{n}^{x}}{g_{n}^{x}}\;+\;\frac{1}{M}\sum_{m\;=\;1}^{M}\frac{s_{m}^{y}}{g_{m}^{y}}\;+\;\frac{1}{K}\sum_{k\;=\;1}^{K}\frac{s_{k}^{z}}{g_{k}^{z}}\right]\\ s.t\left\{\begin{array}{l} \sum_{i\;=\;1}^{I}\lambda_{i}^{t}y_{im}^{t}\;-\;s_{m}^{y}\;=\;y_{im}^{t},\forall m;\\ \sum_{i\;=\;1}^{I}\lambda_{i}^{t}z_{ik}^{t}\;+\;s_{k}^{z}\;=\;z_{ik}^{t},\forall k;\\ \sum_{i\;=\;1}^{I}\lambda_{i}^{t}x_{in}^{t}\;+\;s_{n}^{x}\;=\;x_{in}^{t},\forall n; \end{array}\right.\\ \lambda_{i}^{t}\;\geq \;0,\forall i;s_{n}^{x}\;\geq \;0,\forall n;s_{m}^{y}\;\geq \;0,\forall m;s_{k}^{z}\;\geq \;0,\forall k. \end{array}$$

where $S_{v}^{t}$ is the directional distance function; ($x_{i}^{t}$, $y_{i}^{t}$, $z_{i}^{t}$), ($g^{x}$, $g^{y}$, $g^{z}$), ($s_{n}^{x}$, $s_{m}^{y}$, $s_{k}^{z}$) denote the vector, direction vector, and slack vector of inputs, desired outputs, and undesired outputs, respectively, for *i* city in period *t*. According to Equation (3), the inefficiency value of GTFP can be calculated and converted to the efficiency value, based on the SBM-DDF theorem.

In this paper, 279 prefecture-level cities in China are selected for the study, and the sample interval is from 2006 to 2019. The input, desired output, and non-desired output indicators are described as follows: (1) input indicators. Input indicators include labor, land, energy, and capital inputs. Labor input is reflected in the number of employees in the municipal area; land input is reflected in the built-up area in the municipal area; energy input is reflected in the global stable-night-light value, following the practice of Wu et al. [56]; capital input is measured using the perpetual inventory method, and was deflated by 2006 as the base period to obtain. (2) Expected output-indicator. The expected output-indicator is reflected in the GDP of each region, and the original data are converted, with 2006 as the base period. (3) Undesired output-indicators. Undesired output-indicators are reflected in industrial emissions of the three wastes, including industrial-wastewater discharge, industrial sulfur dioxide emissions, and industrial soot emissions. Table 1 shows the input–output table for green total-factor productivity.

Taking into consideration the fact that the measured GTFP is a chain index, referring to the study of Ma et al. [57], the GTFP is converted into a fixed-base index to represent the cumulative trend of green total-factor productivity. Specifically, using the GTFP for 2006 as 1, the actual GTFP for 2007 is the cumulative multiplication of the GTFP for the current year and the GTFP for 2006, and so on.

#### 4.2.2. Explanatory Variable

The explanatory variables in this paper include formal environmental regulation (FER) and informal environmental regulation (IER). Using the approach of Javorcik et al. [58], the inverse of the ratio of total industrial wastewater, gas, and waste emissions to the gross industrial product, was chosen to represent the formal environmental regulation. Informal environmental regulation is measured by using three indicators, namely, income level, population density, and education level, and calculated using the entropy method [59]. Income level is expressed as the per capita disposable income of urban workers, population density is expressed as the population density of the municipal district, and education level is expressed as the ratio of the number of colleges and universities to the total population at the end of the year.

#### 4.2.3. Control Variable

Green total-factor productivity is influenced by many factors other than environmental regulation. In order to reduce the error of the empirical evidence, this paper proposes the selection of the level of industrial structure (INS), the degree of government intervention (GOV), fiscal decentralization (FIS), the level of openness (OPEN), the level of financial development (FIN), and the level of foreign investment (INVES), as control variables. Among these, the level of industrial structure (INS) is the ratio of the output value of the tertiary sector to the output value of the secondary sector. The degree of government intervention (GOV) is the ratio of municipal-budget expenditure to the gross regional product. Fiscal decentralization (FIS) is the ratio of fiscal revenues to total fiscal expenditures. The level of openness (OPEN) is the ratio of total import and export trade to GDP. The level of financial development (FIN) is the ratio of the sum of the balance of deposits and loans to GDP at the end of the year. The foreign investment level (INVES) is represented by the logarithm of the amount of foreign investment.

### 4.3. Data Sources and Descriptive Statistics

The data selected for this paper are from 2007 to 2019 for 279 prefecture-level cities in China. The data for the environmental-pollution category are from the *China Environmental Statistical Yearbook*, the data for the energy category are from the *China Energy Statistical Yearbook*, and the data for other individual variables are from the *China City Statistical Yearbook* and the *China Regional Statistical Yearbook*. The missing values of relevant indicators in the yearbook are filled in by interpolation. The descriptive statistics of the main variables are shown in Table 2, below. From Table 2, the difference between the maximum and minimum values of GTFP, FER, and IER is large, indicating that the regional differences in green total-factor productivity and double environmental-regulation levels in China are relatively obvious, and the regional imbalance is more apparent. The standard deviation of INVES is smaller than the mean value, indicating that the dispersion is reduced after taking the log lag, and it is more reasonable to take the measure in the form of a logarithm, for the variable.

## 5. Empirical Analysis

### 5.1. GTFP in China

**Figure 2:**
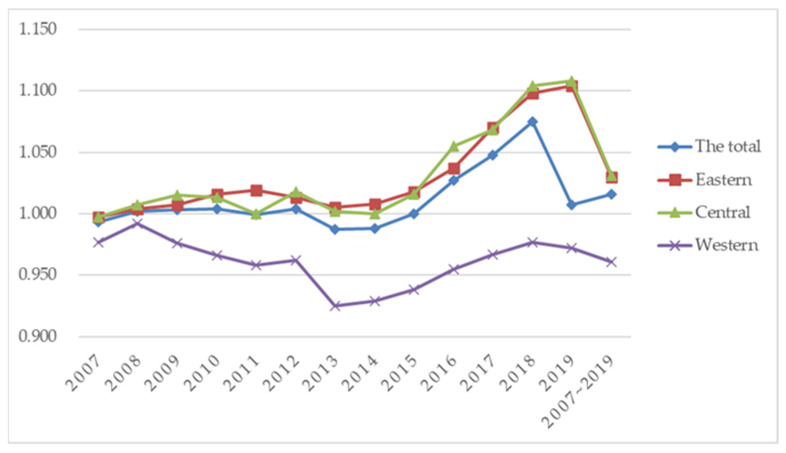
Green total-factor productivity (GTFP) in different regions of China.

In this paper, we divide cities into eastern, central, and western regions for comparison, and analyze the changes of GTFP in the regions where cities are located, as shown in Figure 2. Figure 2 shows that “strong in the east and weak in the west” is the main characteristic of GTFP changes in the three regions. The range of GTFP fluctuates between 0.9 and 1.15, with a relatively small variation, but there is a regional-crossover variation between different years. Up to 2019, the central region is higher than the eastern region, and both are above the total level, while the western region is below the total-level line. The change of GTFP in the eastern and central regions is roughly in line with the total level, while the change of GTFP in the western region has a lag, slightly slower than the eastern and central regions.

This is probably because the eastern region is strategically located, with a high level of scientific and technological innovation, and a concentration of high-tech industries, which are more capable of promoting the upgrading of the green-industry structure, and driving up the GTFP. The western region is located inland, and lacks the necessary talent and technology to develop a green economy, leading to the economic development of the industry being mostly about traditional heavy industries and manufacturing, with high consumption, high pollution, and high emissions. Therefore, compared with the eastern and central regions, the development of GTFP in the western region has a certain lag.

### 5.2. Spatial-Autocorrelation Test and Selection of Spatial-Econometric Model

#### 5.2.1. Spatial-Autocorrelation Test

This paper uses the global Moran index to test the spatial autocorrelation between dual environmental regulations and green total-factor productivity. Table 3 shows the global Moran index for each variable. The Moran-index values for dual environmental regulations and green total-factor productivity from 2007 to 2019 are significantly positive, indicating a significant spatial autocorrelation among them. Therefore, the spatial-econometric model is suitable for the study of this paper.

#### 5.2.2. Selection of Spatial-Econometric Model

In order to further investigate which spatial-econometric model should be used, the LM, LR, and Wald tests are used to conclude that the spatial Durbin model should be used. Table 4 shows the results of the LM, LR, and Wald tests. The four tests of LM significantly reject the original hypothesis at the 1% level, indicating that the spatial-panel model is more suitable for the study of this paper. The LR and Wald tests for the spatial-lag and spatial-error models are both significant at the 1% level, indicating that the spatial Durbin model cannot be reduced to a spatial-lag model and a spatial-error model. The LR tests for time and space also indicate that the spatial Durbin model has both time and space fixed-effects. Meanwhile, it is more appropriate to use a fixed-effect model with the Hausman test. Therefore, combining the above test results, this paper uses a double-fixed spatial Durbin model for regression analysis.

### 5.3. Benchmark Regression Results

Table 5 reports the results of the estimation of dual environmental-regulation on green total-factor productivity. For comparative analysis, the regression results of fixed effects (FE) and the spatial Durbin model (SDM) are presented in the table. Columns (1) and (2) present the results of an ordinary panel estimation of the effect of dual environmental-regulation on green total-factor productivity. Columns (3) and (4) present the estimation results of the spatial Durbin model for double fixation.

From the spatial-autoregressive coefficients, the two spatial-autoregressive coefficients of the spatial Durbin model are 0.631 and 0.496, respectively, and both pass the 1% significance-level test, indicating that there is a significant spatial correlation of GTFP, further indicating that the model used in this paper is reasonable.

In terms of formal environmental regulation, the results in Column (3) show that the primary coefficient of FER is 0.116 and the quadratic coefficient of FER^2^ is −0.005, with a negative quadratic coefficient, both of which are significant at the 1% level, indicating that formal environmental regulation has a significant inverted-U-shaped relationship on GTFP, and hypothesis H1 is verified. In terms of informal environmental regulation, the primary coefficient of IER in Column (4) is −0.180 and the quadratic coefficient of IER2 is 0.417, with a positive quadratic coefficient, both of which pass the 1% significance-level test, indicating that informal environmental regulation has a significant U-shaped relationship on GTFP, and that hypothesis H2 is verified.

In terms of control variables, INS and INVES have a positive effect on GTFP, and can improve it. The regression coefficient of FIS does not pass the significance test, indicating that FIS is not the main driver of GTFP. The remaining three control variables, GOV, OPEN, and FIN, have negative effects on GTFP, indicating that excessive intensity of government regulation on the environment does not increase green total-factor productivity; simply expanding the total import- and export-trade does not guarantee a rise in the level of green development, and polluting enterprises may also obtain funds through financial channels, reducing the financial support for environmental-protection enterprises, and leading to a decline.

### 5.4. Analysis of Spatial Effects

Although there is a significant effect of dual environmental-regulation on green total-factor productivity in Table 5, it is not representative of the marginal effect of dual environmental-regulation on green total-factor productivity. To further analyze the spatial-spillover effects of each variable, this paper decomposes the spatial effects into direct and indirect effects. Table 6 shows the results of the spatial-effect decomposition. In terms of direct effects, formal environmental regulation has a significant inverted-U-shaped effect on GTFP in local areas, while informal environmental regulation has a significant U-shaped effect. It shows that for the local region, during the initial stage of the government making environmental-regulation policies, it will force enterprises to make technological innovations, and formal environmental regulation will positively affect GTFP. With increasingly stringent environmental-regulation policies and excessive environmental pressure on enterprises, formal environmental regulation will instead hinder GTFP. When public awareness of environmental protection is weak, informal environmental regulation plays a limited role, and is not conducive to the improvement of GTFP. As public awareness of environmental protection continues to increase, and channels for participation in environmental protection continue to improve, informal environmental regulation becomes more powerful for environmental protection, which will promote GTFP. In terms of indirect effects, the coefficient of the primary term of formal environmental regulation on GTFP in neighboring regions is significantly negative, while the coefficient of the second term is positive but insignificant, and hypothesis H3 is partially verified. The possible reason is that the increase in the intensity of formal environmental regulation in the local regions will force the polluters to move into the neighboring regions, resulting in a decrease in GTFP in the neighboring areas. In addition, informal environmental regulation also has a significant U-shaped effect on GTFP, and hypothesis H3 is confirmed. Informal environmental regulation inhibits GTFP in neighboring regions before promoting it.

### 5.5. Spatial-Heterogeneity Analysis

We consider that differences in resource endowments and environmental pressures across regions will lead to differences in the intensity of environmental regulations, which will result in heterogeneity in the response of GTFP to dual environmental-regulation. Therefore, on the one hand, this paper divides the 279 prefecture-level cities into two regions, the eastern region and the central-western region. On the other hand, cities are classified according to the variability of economic development of each city, and, drawing on Li et al. [60], first-tier cities, new first-tier cities, and second-tier cities are classified as first-class cities, and third-tier cities, four-tier cities, and fifth-tier cities are classified as second-class cities. The results are shown in Table 7.

From a regional perspective, the direct-effect coefficients of both formal and informal environmental regulations in the eastern region have significant inverted U-shaped and U-shaped effects on GTFP, confirming hypotheses H1 and H2. However, there are variations in the indirect effects, with a significantly negative coefficient on the primary term, and a positive but insignificant coefficient on the quadratic term for formal environmental regulation. The coefficients of the primary and quadratic terms of informal environmental regulation are not significant. This indicates that polluting enterprises in the eastern region play the role of “pollution transfer paradise” in the transfer process, and environmental regulation is not the main driver for improving GTFP in neighboring regions. The direct and indirect coefficients of formal and informal environmental regulations in the central-western regions have inverted-U-shaped and U-shaped effects on GTFP, but the individual coefficients are not significant. The reason is probably that the level of environmental regulation in the central-western regions is relatively lagging, and the effect on GTFP is not obvious.

In terms of city classification, the direct-effect coefficients of formal environmental regulation have a significant inverted-U-shaped effect on GTFP in first-class and second-class cities, while informal environmental regulation has a U-shaped effect, but not significant. Whereas the indirect-effect coefficients differ, the indirect-effect coefficients of dual environmental-regulation in first-class cities have a significant effect on GTFP in neighboring areas, and the indirect-effect coefficients of formal environmental regulations in second-class cities are not significant. The possible reason is that the implementation of environmental policies in first-class cities, which have a higher level of economic development, may generate demonstration-learning effects and spatial-spillover effects on neighboring areas. In contrast, second-class cities are less developed in terms of transportation and economic development, and the implementation of their environmental policies may not have exemplary learning effects or significant spatial-spillover effects on neighboring areas.

### 5.6. Robustness Test

In order to increase the reliability of the research results, this paper performs robustness examinations using two methods: first, using the SBM-GML method to measure GTFP; second, replacing the weight matrix. The results of the robustness test are shown in Table 8. The significance and sign of the dual environmental-regulation coefficients are generally consistent with the results of the previous empirical analysis, indicating that the results of this paper are robust and reliable.

## 6. Further Research: Moderating and Threshold-Effects of Green-Technology Innovation

The previous paper verified that dual environmental-regulations not only have direct effects on green total-factor productivity from an independent perspective, but also generate spatial-spillover effects. Therefore, can green innovation levels have a contribution effect on the process of dual environmental-regulation affecting green total-factor productivity? Meanwhile, can different levels of green technological-innovation produce a threshold effect in the process of dual environmental-regulation affecting green total-factor productivity? This section will empirically analyze the moderating role and threshold role of green technological-innovation within dual environmental-regulation affecting green total-factor productivity from a coordinated-interaction perspective. Green Technology-Innovation (GTI), expressed in terms of the number of green-invention patent-applications, and green-patent data, are from the State Intellectual Property Office.

### 6.1. Analysis of Moderating Effect

Firstly, the moderating effect of green technological-innovation (GTI) is tested by introducing the interaction term and spatial-lag term of GTI and dual environmental-regulation, based on Equations (1) and (2), respectively. The specific models are set as follows:

(4) $$\begin{array}{ll} {\mathrm{GTFP}}_{it}\;= & \;\rho \sum_{j\;=\;1,j\;\neq \;i}^{n}W_{ij}{\mathrm{GTFP}}_{it}\;+\;\delta_{1}{\mathrm{FER}}_{it}\;+\;\delta_{2}{{\mathrm{FER}}^{2}}_{it}\;+\;\delta_{3}{\mathrm{FER}}_{it}\;\times \;GTI\;+\;\delta_{4}{{\mathrm{FER}}^{2}}_{it}\;\times \;GTI\;+\;\gamma {\mathrm{Control}}_{it}\;+\;\theta \sum_{j\;=\;1,j\;\neq \;i}^{n}W_{ij}\\ & ({\mathrm{FER}}_{it}\;+\;{{\mathrm{FER}}^{2}}_{it}\;+\;{\mathrm{FER}}_{it}\;\times \;GTI\;+\;{{\mathrm{FER}}^{2}}_{it}\;\times \;GTI\;+\;{\mathrm{Control}}_{it})\;+\;\mu_{i}\;+\;\eta_{t}\;+\;\varepsilon_{it} \end{array}$$

(5) $$\begin{array}{ll} {\mathrm{GTFP}}_{it}\;= & \;\rho \sum_{j\;=\;1,j\;\neq \;i}^{n}W_{ij}{\mathrm{GTFP}}_{it}\;+\;\delta_{5}{\mathrm{IER}}_{it}\;+\;\delta_{6}{{\mathrm{IER}}^{2}}_{it}\;+\;\delta_{7}{\mathrm{IER}}_{it}\;\times \;GTI\;+\;\delta_{8}{{\mathrm{IER}}^{2}}_{it}\;\times \;GTI\;+\;\gamma {\mathrm{Control}}_{it}\;+\;\theta \sum_{j\;=\;1,j\;\neq \;i}^{n}W_{ij}\\ & ({\mathrm{IER}}_{it}\;+\;{{\mathrm{IER}}^{2}}_{it}\;+\;{\mathrm{IER}}_{it}\;\times \;GTI\;+\;{{\mathrm{IER}}^{2}}_{it}\;\times \;GTI\;+\;{\mathrm{Control}}_{it})\;+\;\mu_{i}\;+\;\eta_{t}\;+\;\varepsilon_{it} \end{array}$$

The results of the moderating effect are shown in Table 9. In terms of the moderating effect of green-technology innovation on formal environmental regulation and green total-factor productivity, the coefficient of the interaction term between the primary term of formal environmental regulation and green-technology innovation (FER × GTI) is negative in both direct and spatial-spillover effects, while the coefficient of the interaction term between the quadratic term of formal environmental regulation and green-technology innovation (FER^2^ × GTI) is positive in direct and spatial-spillover effects, indicating that green-technology innovation negatively moderates the effect of formal environmental regulation on green total-factor productivity. Accordingly, green technological-innovation attenuates the positive effect of formal environmental regulation on green total-factor productivity in the first half of the inverted U-shape, and the negative effect in the second half in the local regions and neighboring regions. In the initial stage of environmental-regulation policy-formulation, enterprises with high technological innovation tend to enhance their profits, which weakens the positive impact of formal environmental regulation on green total-factor productivity in the first half. When the environmental-regulation policy tends to mature, both in regions with a high or low level of green technological-innovation, the environmental-regulation policy can induce firms to reduce energy consumption and emissions, which attenuates the negative effect in the second half.

In terms of the moderating effect of green-technology innovation on informal environmental regulation and green total-factor productivity, the coefficient of the interaction term between the primary term of informal environmental regulation and green-technology innovation (IER × GTI) is positive in the direct effect and negative in the spatial-spillover effect, while the coefficient of the interaction term between the quadratic term of informal environmental regulation and green-technology innovation (FER^2^ × GTI) is negative in the direct effect and positive in the spatial-spillover effect. It is shown that green-technology innovation negatively moderates the effect of informal environmental regulation on green total-factor productivity in local regions, and positively moderates the effect of informal environmental regulations on green total-factor productivity in neighboring regions. It is possible that for neighboring areas, at the beginning of informal environmental regulation, local areas relocate polluting firms to neighboring areas, to rapidly meet the public demand for environmental protection, which leads to the decline in environmental quality in neighboring areas and aggravates the first half of the negative effect of informal environmental regulation on green total-factor productivity. As the public gradually pays attention to environmental issues, the level of green technological-innovation by enterprises and individuals increases, which enhances the second half of the positive impact of informal environmental regulation on green total-factor productivity in neighboring areas.

### 6.2. Analysis of Threshold Effect

Secondly, we consider that green-technology innovation may affect the correlation between dual environmental-regulation and green total-factor productivity, resulting in a nonlinear impact relationship between them. Based on this, this paper further constructs a threshold-effect model according to Hansen [61], to analyze the threshold effect of different levels of green technological-innovation for dual environmental regulation affecting green total-factor productivity. The threshold models are set as follows:

(6) $$\begin{array}{ll} {\mathrm{GTFP}}_{it} & \;=\;\alpha_{0}\;+\;\alpha_{1}FER\;\times \;I(GTI\;\leq \;{ϒ}_{1})\;+\;\alpha_{2}{\mathrm{FER}}_{it}\;\times \;I({ϒ}_{1}\;<\;GTI\;\leq \;{ϒ}_{2})\;+\;\alpha_{3}{\mathrm{FER}}_{it}\;\times \;I(GTI\;>\;{ϒ}_{2})\\ & \;+\;\gamma {\mathrm{Control}}_{it}\;+\;\varepsilon_{it} \end{array}$$

(7) $$\begin{array}{ll} {\mathrm{GTFP}}_{it} & \;=\;\alpha_{0}\;+\;\alpha_{4}IER\;\times \;I(GTI\;\leq \;{ϒ}_{1})\;+\;\alpha_{5}{\mathrm{IER}}_{it}\;\times \;I({ϒ}_{1}\;<\;GTI\;\leq \;{ϒ}_{2})\;+\;\alpha_{6}{\mathrm{IER}}_{it}\;\times \;I(GTI\;>\;{ϒ}_{2})\\ & \;+\;\gamma {\mathrm{Control}}_{it}\;+\;\varepsilon_{it} \end{array}$$

where the threshold variable is green technological-innovation (GTI); I(·) denotes the indicator function; Υ is the threshold value to be estimated; α is a constant term, and α_1_, α_2_, …, α_6_ are the magnitudes of the coefficients of dual environmental-regulation at different threshold intervals.

The threshold and statistics are estimated using 500 iterations of bootstrap estimation, and the specific test results are shown in Table 10. When the independent variable is formal environmental regulation (FER), there is a single threshold for green-technology innovation, with a threshold value of 0.0245. When the independent variable is informal environmental regulation (IER), there is a double threshold for green-technology innovation, with a threshold value of 0.0031 and 0.2861.

The results of the threshold regression are shown in Table 11. From formal environmental regulation (FER), when GTI ≤ 0.0245, the coefficient of FER is 0.290; when GTI > 0.0245, the coefficient of FER is 0.589. From informal environmental regulation (IER), when GTI ≤ 0.0031, the coefficient of IER is 0.075; when 0.0031 < GTI ≤ 0.286, the coefficient of IER is 0.232; when GTI > 0.2861, the coefficient is 0.551. All the above coefficients are significantly positive. This shows that the dual environmental-regulations always positively promote green total-factor productivity as the level of green innovation increases, but the promotion effect varies across intervals, and shows a gradual increase. This may be due to the fact that the cumulative effect formed by green technological-innovation will play a more obvious role when the level of green innovation reaches a certain critical point [62], and it shows a significant promotion effect, regardless of the interval.

## 7. Conclusions, Discussion and Policy-Recommendations

### 7.1. Conclusions

Based on the panel data of 279 prefecture-level cities in China from 2007 to 2019, this paper measures the index of GTFP using the SBM-DDF method, and employs the spatial Durbin model to examine the direct effect, spatial-spillover effect, and interregional heterogeneity of dual environmental-regulation on GTFP. Moreover, we further analyze the moderating effect and threshold effect of green technological-innovation in the process of dual environmental-regulation affecting GTFP. The main conclusions are as follows: (1) there is an inverted-U-shaped relationship between formal environmental regulation and GTFP, and formal environmental regulation has an inverted U-shaped effect on GTFP in local regions, while its spatial-spillover effect is not significant. The effect of formal environmental regulations on GTFP shows heterogeneity, and the direct effect of formal environmental regulation in eastern, central-western regions and first-class and second-class cities has a significant inverted-U-shaped effect, but the spatial-spillover effect is significantly different. (2) There is a U-shaped relationship between informal environmental regulation and GTFP, specifically, informal environmental regulation not only has a U-shaped impact on GTFP in local regions, but also has a U-shaped spatial-spillover effect. The effect of informal environmental regulation on GTFP also shows heterogeneity. The direct effect of informal environmental regulation in the eastern region has a significant U-shaped effect, while the coefficients of the direct effect in the rest of the regions and cities are not significant, and the spatial-spillover effects of informal environmental regulation also vary significantly among regions. (3) Green-technology innovation is an important moderating variable for the impact of dual environmental-regulation on GTFP. Green innovation negatively moderates the inverted-U-shaped relationship between formal environmental regulation and GTFP in local and neighboring regions. However, green-technology innovation negatively moderates the U-shaped relationship between informal environmental regulation and GTFP in local regions, but positively moderates the relationship in neighboring regions. (4) There is a significant green-technology innovation-threshold effect on dual environmental regulation affecting GTFP, and as the level of green-technology innovation increases, the promotion effect of dual environmental-regulation on GTFP gradually improves.

### 7.2. Discussion

The contributions of this paper are mainly in the following two aspects: first, the data of Chinese prefecture-level cities are used to explore the effects of formal and informal environmental regulations on GTFP, thus verifying the reliability of some of the literature findings. Second, this paper not only analyzes the effects of dual environmental-regulations on GTFP, but also discusses the heterogeneity-spillover effects and heterogeneity across regions. Furthermore, the moderating effects and threshold effects of green-technology innovation are analyzed, which makes the research results more comprehensive.

The research on environmental regulation and green total-factor productivity is a popular topic. In the existing studies, Bartik stated that several measures of environmental regulations in the United States tend to shift polluting industries from states where marginal pollution-damage is low, to states where this damage is high [63]. Similarly, the study of Jorgenson and Wilcoxen confirmed this statement [64]. In addition, the Porter hypothesis provides a fresh perspective on the relationship between environmental quality and economic development. The study of Xie et al. found evidence to support the “strong” version of the Porter hypothesis, that reasonable stringency of environmental regulations may enhance green productivity [65]. However, since different regions in China are undergoing different stages of development, some regions might have matured to the middle to the higher-income level of the developmental stage where environmental concerns outweigh small economic-growth. That might have explained the inverted-U-shape effect. Our study advances the current research by dividing environmental regulation into formal environmental regulation, represented by the government, and informal environmental regulation, represented by the public, and by studying the moderating effect and threshold effect of green-technology innovation. This helps us to find the institutional factors for the validity of the Porter hypothesis, and supports us in exploring effective paths to improve green total-factor productivity and green development, in China.

The limitations of this study include: firstly, the indicator system for constructing GTFP in this paper is not rich, and the considered dimensions are not comprehensive. There may be slight differences from reality. In the following research, we will explore the methods of measuring GTFP in a deeper way and from more perspectives, further enrich the research indicators and methodologies, and broaden the ideas. Secondly, the regression results in this paper may also be affected by other environmental factors (green-energy production, etc.). However, this paper’s research data are for Chinese prefecture-level cities, and there are large amounts of missing annual environmental-data for some cities, to complete the regression results. The next step of the study will explore an effective data-completion method, to complete the missing values.

### 7.3. Policy Recommendations

Based on the theoretical and empirical results, this paper puts forward the following recommendations:

Firstly, the government should implement a more precise environmental regulation policy, and determine the appropriate intensity of environmental regulation. High-intensity formal environmental regulation can promote green total-factor productivity in a short period, but it will also increase the costs and burdens of enterprises. Therefore, while improving the relevant environmental laws and regulations, the government should implement different levels of environmental-regulation intensity standards, according to regional- and industrial-structure differences, increase the penalties for heavy polluters, turn the effect of regulation into a positive effect as soon as possible, and give certain subsidies and support to environmental-protection enterprises, to create a favorable environment for them, which will realize the green- and coordinated-development of environmental protection and enterprises.

Secondly, environmental-protection departments should pay more attention to informal environmental regulation, and guide the public to participate rationally in environmental protection. On the one hand, environmental-protection departments should strengthen the publicity of energy conservation and emission reduction, improve the public environmental-protection supervision mechanism and broaden public supervision-channels, and enhance the enthusiasm for public participation in environmental supervision. On the other hand, the government should provide forward-looking environmental education to the public, cultivate “green and high quality” talents, and advocate rational participation in environmental protection.

Thirdly, we should continue to increase the investment in green-technology innovation and reduce the risk of green-technology innovation in enterprises. On the one hand, we should give sufficient financial-support and tax concessions and exemptions to enterprises’ green-technology research and development, bring new vitality into enterprises’ green-technology innovation, increase scientific and technological research-efforts, promote enterprises’ development in the direction of intelligence and greening, and cultivate various new industries, such as ecological industries and green industries. On the other hand, we should introduce relevant experts and scholars into the field of green-technology innovation, combine with universities and research institutes to cultivate high-tech talents, and provide “hardware” technical-support for promoting green development.

Finally, we should strengthen regional cooperation among regions, and explore methods to improve the cooperation- and coordination-mechanism of dual environmental-regulation and green-technology innovation in various regions, to achieve a “win-win” situation.

## Figures and Tables

**Table 1:** Indicator index system for GTFP in China.

Variable	Indicator	Index	Definition
GTFP	Input	Labor	Number of employees in the municipal area
		Land	The built-up area in the municipal area
		Energy	Global stable-night-light value
		Capital	Measured by the perpetual inventory method
	Expected Output	Economic output	Real GDP of each region
	Undesired Output	Environmental pollution	Industrial-wastewater discharge
			Industrial sulfur dioxide emissions
			Industrial soot emissions

**Table 2:** Descriptive statistics of the main variables.

Variable Category	Variable Symbol	Number of Observations	Mean Value	Standard Deviation	Minimum Value	Maximum Value
Explained variable	GTFP	3627	1.016	0.130	0.206	1.877
Explanatory variable	FER	3627	0.056	0.381	4.39 × 10^−5^	17.580
	IER	3627	0.189	0.094	0.024	0.694
Control variable	INS	3627	1.081	0.663	0.0943	6.533
	GOV	3627	17.040	10.740	1.021	270.200
	FIS	3627	0.483	0.361	0.0544	6.131
	OPEN	3627	0.194	0.368	1.34 × 10^−5^	8.168
	FIN	3627	3.029	1.740	0.213	62.890
	INVES	3627	11.660	2.027	3.008	16.830

**Table 3:** The Global Moran’s index of main variables from 2007 to 2019.

Year	GTFP	FER	IER
2007	0.013 ***	0.034 ***	0.011 ***
2008	0.017 ***	0.035 ***	0.016 ***
2009	0.006 *	0.028 ***	0.016 ***
2010	0.013 ***	0.028 ***	0.015 ***
2011	0.007 **	0.023 ***	0.010 **
2012	0.008 **	0.028 ***	0.009 **
2013	0.022 ***	0.040 ***	0.011 ***
2014	0.015 ***	0.044 ***	0.009 **
2015	0.018 ***	0.060 ***	0.014 ***
2016	0.020 ***	0.072 ***	0.014 ***
2017	0.016 ***	0.062 ***	0.013 ***
2018	0.041 ***	0.024 ***	0.015 ***
2019	0.027 ***	0.011 ***	0.015 ***

Note: *** *p* < 0.01, ** *p* < 0.05, * *p* < 0.1.

**Table 4:** LM, LR, and Wald tests.

Test Statistic	Statistical Value	*p* Value
LM-test-lag	713.346	0.000
Robust LM-test-lag	62.309	0.000
LM-test-error	820.611	0.000
Robust LM-test-error	169.573	0.000
LR-test-lag	101.48	0.000
Wald-test-lag	102.56	0.000
LR-test-error	74.99	0.000
Wald-test-error	74.23	0.000

**Table 5:** Benchmark-regression results.

Variable	(1)	(2)	(3)	(4)
	FE		SDM	
FER	0.091 ***		0.116 ***	
	(9.646)		(12.330)	
FER^2^	−0.004 ***		−0.005 ***	
	(−6.086)		(−8.479)	
IER		−0.207 **		−0.180 **
		(−2.509)		(−2.271)
IER^2^		0.401 ***		0.417 ***
		(2.788)		(3.028)
INS	0.022 ***	0.020 ***	0.017 ***	0.016 ***
	(5.115)	(4.580)	(4.266)	(3.865)
GOV	−0.000 **	−0.000 **	−0.000 **	−0.000 **
	(−2.073)	(−2.076)	(−2.378)	(−2.302)
FIS	−0.013	−0.014	−0.013	0.009
	(−0.664)	(−0.680)	(−0.619)	(0.438)
OPEN	0.002	−0.011 *	−0.004	−0.012 *
	(0.302)	(−1.751)	(−0.620)	(−1.935)
FIN	−0.014 ***	−0.014 ***	−0.015 ***	−0.015 ***
	(−11.954)	(−11.939)	(−13.626)	(−13.022)
INVES	−0.000	0.000	0.002	0.003 *
	(−0.056)	(0.186)	(1.106)	(1.959)
Constant	1.017 ***	1.038 ***		
	(51.674)	(48.222)		
W × FER			−0.369 ***	
			(−4.661)	
W × FER^2^			0.006	
			(1.087)	
W × IER				−4.414 ***
				(−3.973)
W × IER^2^				11.050 ***
				(4.947)
*p*			0.631 ***	0.496 ***
N	3627	3627	3627	3627
Individual fixed	YES	YES	YES	YES
Time fixed	YES	YES	YES	YES

Note: *** *p* < 0.01, ** *p* < 0.05, * *p* < 0.1. The t value is in parentheses.

**Table 6:** Decomposition of spatial effect.

Variable	Direct Effect	Indirect Effect	Total Effect
FER	0.113 ***	−0.853 ***	−0.739 ***
FER^2^	−0.005 ***	0.007	0.002
IER	−0.207 ***	−9.398 ***	−9.605 ***
IER^2^	0.478 ***	23.486 ***	23.963 ***
Control variables	YES	YES	YES
Individual fixed	YES	YES	YES
Time fixed	YES	YES	YES

Note: *** *p* < 0.01.

**Table 7:** Analysis of spatial heterogeneity.

	Eastern Cities	Central-Western Cities	First-Class Cities	Second-Class Cities
Direct-FER	0.105 ***	0.420 ***	0.091 ***	0.327 ***
Direct-FER^2^	−0.004 ***	−0.546 ***	−0.004 ***	−0.199 ***
Direct-IER	−0.688 ***	−0.072	−0.189	−0.133
Direct-IER^2^	1.301 ***	0.275 **	0.123	0.256
Indirect-FER	−0.289 ***	4.200 **	−0.161 ***	0.738
Indirect-FER^2^	0.005	−7.313	0.004 *	−2.036
Indirect-IER	−1.100	−4.377 **	5.098 ***	−14.980 ***
Indirect-IER^2^	1.272	14.382 ***	−10.208 ***	31.681 ***
Total-FER	−0.185 ***	4.619 **	−0.069 *	1.066
Total-FER^2^	0.001	−7.859	−0.000	−2.235
Total-IER	−1.788	−4.449 **	4.908 ***	−15.113 ***
Total-IER^2^	2.573	14.658 ***	−10.085 ***	31.937 ***
Control variables	YES	YES	YES	YES
Individual fixed	YES	YES	YES	YES
Time fixed	YES	YES	YES	YES

Note: *** *p* < 0.01, ** *p* < 0.05, * *p* < 0.1.

**Table 8:** Robustness test.

Variable	(1)	(2)	(3)	(4)	(5)	(6)
	Remeasurement of GTFP		0–1 Weighting Matrix		Economic and Geographic Distance Weighting Matrix	
FER	0.334 ***		0.117 ***		0.121 ***	
	(7.250)		(12.420)		(12.841)	
FER^2^	−0.011 ***		−0.005 ***		−0.005 ***	
	(−4.094)		(−8.293)		(−8.708)	
IER		−2.954 ***		−0.241 ***		−0.202 **
		(−7.807)		(−3.008)		(−2.508)
IER^2^		4.475 ***		0.446 ***		0.414 ***
		(6.795)		(3.227)		(2.986)
Control variables	YES	YES	YES	YES	YES	YES
Individual fixed	YES	YES	YES	YES	YES	YES
Time fixed	YES	YES	YES	YES	YES	YES

Note: *** *p* < 0.01, ** *p* < 0.05. The t value is in parentheses.

**Table 9:** Analysis of the moderating effect of green-technology innovation.

Variable	Direct Effect	Indirect Effect	Total Effect	Direct Effect	Indirect Effect	Total Effect
FER	0.565 ***	12.487 *	13.052 *			
	(5.454)	(1.664)	(1.733)			
FER^2^	−0.105 ***	−6.406 **	−6.511 **			
	(−2.883)	(−2.304)	(−2.330)			
FER × GTI	−0.264 ***	−2.540	−2.804			
	(−3.087)	(−0.415)	(−0.455)			
FER^2^ × GTI	0.071 ***	4.068 **	4.140 **			
	(3.008)	(2.204)	(2.231)			
GTI	−0.695 ***	28.167 ***	27.472 ***	−0.695 ***	28.167 ***	27.472 ***
	(−3.250)	(3.453)	(3.361)	(−3.250)	(3.453)	(3.361)
IER				−0.133	−9.404 ***	−9.537 ***
				(−1.614)	(−3.146)	(−3.188)
IER^2^				0.280 *	24.713 ***	24.993 ***
				(1.902)	(3.700)	(3.731)
IER × GTI				4.560 ***	−142.060 ***	−137.501 ***
				(3.888)	(−3.192)	(−3.083)
IER^2^ × GTI				−5.593 ***	149.397 **	143.805 **
				(−3.418)	(2.550)	(2.447)
Control variables	YES	YES	YES	YES	YES	YES
Individual fixed	YES	YES	YES	YES	YES	YES
Time fixed	YES	YES	YES	YES	YES	YES

Note: *** *p* < 0.01, ** *p* < 0.05, * *p* < 0.1. The t value is in parentheses.

**Table 10:** The test of a threshold effect.

Independent Variable	Model	F-Stat	Prob	Critical Value			Threshold Value	95% Confidence Interval
				10%	5%	1%		
FER	Single	131.22	0.000	38.187	46.757	74.855	0.0245	[0.0226, 0.0247]
	Double	21.58	0.170	26.371	35.109	56.324	—	—
IER	Single	90.96	0.000	36.367	43.011	53.605	0.0031	[0.0029, 0.0032]
	Double	74.25	0.006	32.356	41.932	64.390	0.2861	[0.2742, 0.2923]
	Triple	24.48	0.489	44.238	56.561	84.378	—	—

**Table 11:** The estimation results of the threshold-regression model.

Variable	(1)	(2)
FER (GTI ≤ 0.0245)	0.290 ***	
	(10.136)	
FER (GTI > 0.0245)	0.589 ***	
	(9.471)	
IER (GTI ≤ 0.0031)		0.075 *
		(1.908)
IER (0.0031 < GTI ≤ 0.2861)		0.232 ***
		(6.587)
IER (GTI > 0.2861)		0.551 ***
		(11.812)
Control variables	YES	YES

Note: *** *p* < 0.01, * *p* < 0.1. The *t* value is in parentheses.

## Data Availability

The data will be available on request.

## References

[1] Ni Y., Chen B., Wang Y. (2020). Financial development, environmental regulation and green total factor productivity—An empirical analysis based on spatial Durbin model. J. Guizhou Univ. Financ. Econ..

[2] Gezhi W., Daming Y. (2019). Mechanisms of environmental regulation on technological innovation and green total factor productivity: The moderating role based on fiscal decentralization. J. Ind. Eng. Eng. Manag..

[3] Gollop F.M., Roberts M.J. (1983). Environmental Regulations and Productivity Growth: The Case of Fossil-fueled Electric Power Generation. J. Polit. Econ..

[4] Gray W.B., Shadbegian R.J. (2003). Plant vintage, technology, and environmental regulation. J. Environ. Econ. Manag..

[5] Pargal S., Wheeler D. (1996). Informal regulation of industrial pollution in developing countries: Evidence from Indonesia. J. Polit. Econ..

[6] Xiong B., Wang R. (2020). Effect of environmental regulation on industrial solid waste pollution in China: From the perspective of formal environmental regulation and informal environmental regulation. Int. J. Environ. Res. Public Health.

[7] Zhao X., Sun B. (2016). The influence of Chinese environmental regulation on corporation innovation and competitiveness. J. Clean. Prod..

[8] Luo Y., Salman M., Lu Z. (2021). Heterogeneous impacts of environmental regulations and foreign direct investment on green innovation across different regions in China. Sci. Total Environ..

[9] Porter M.E., van der Linde C. (1995). Toward a New Conception of the Environment-Competitiveness Relationship. J. Econ. Perspect..

[10] Jaffe A.B., Palmer K. (1997). Environmental regulation and innovation: A panel data study. Rev. Econ. Stat..

[11] Cheng Z.H., Kong S.Y. (2022). The effect of environmental regulation on green total-factor productivity in China’s industry. Environ. Impact. Asses..

[12] Lee C.-C., Zeng M., Wang C. (2022). Environmental regulation, innovation capability, and green total factor productivity: New evidence from China. Environ. Sci. Pollut. Res..

[13] Lena D., Pasurka C.A., Cucculelli M. (2022). Environmental regulation and green productivity growth: Evidence from Italian manufacturing industries. Technol. Forecast. Soc..

[14] Tong L., Chiappetta Jabbour C.J., ben Belgacem S., Najam H., Abbas J. (2022). Role of environmental regulations, green finance, and investment in green technologies in green total factor productivity: Empirical evidence from Asian region. J. Clean. Prod..

[15] Rexhäuser S., Rammer C. (2014). Environmental Innovations and Firm Profitability: Unmasking the Porter Hypothesis. Environ. Resour. Econ..

[16] Yuan B., Xiang Q. (2018). Environmental regulation, industrial innovation and green development of Chinese manufacturing: Based on an extended CDM model. J. Clean. Prod..

[17] Zhan L., Guo P., Pan G. (2022). The effect of mandatory environmental regulation on green development efficiency: Evidence from China. Environ. Sci. Pollut. Res..

[18] Lanoie P., Patry M., Lajeunesse R. (2008). Environmental regulation and productivity: Testing the porter hypothesis. J. Prod. Anal..

[19] Wang Y., Sun X., Guo X. (2019). Environmental regulation and green productivity growth: Empirical evidence on the Porter Hypothesis from OECD industrial sectors. Energy Policy.

[20] Zhao M.L., Liu F.Y., Sun W., Tao X. (2020). The relationship between environmental regulation and green total factor productivity in China: An empirical study based on the panel data of 177 cities. Int. J. Environ. Res. Public Health.

[21] Li Y., Li S. (2021). The Influence Study on Environmental Regulation and Green Total Factor Productivity of China’s Manufacturing Industry. Discrete Dyn. Nat. Soc..

[22] Wang Z.L., Yang Y.Q., Wei Y. (2022). Study on relationship between environmental regulation and green total factor productivity from the perspective of FDI—Evidence from China. Sustainability.

[23] Wang Y., Shen N. (2016). Environmental regulation and environmental productivity: The case of China. Renew. Sustain. Energy Rev..

[24] He Q., Han Y., Wang L. (2021). The impact of environmental regulation on green total factor productivity: An empirical analysis. PLoS ONE.

[25] Luo G., Wang X., Wang L., Guo Y. (2021). The relationship between environmental regulations and green economic efficiency: A study based on the provinces in China. Int. J. Environ. Res. Public Health.

[26] Qiu S., Wang Z., Geng S. (2021). How do environmental regulation and foreign investment behavior affect green productivity growth in the industrial sector? An empirical test based on Chinese provincial panel data. J. Environ. Manag..

[27] Wang L., Yan Y. (2022). Environmental Regulation Intensity, Carbon Footprint and Green Total Factor Productivity of Manufacturing Industries. Int. J. Environ. Res. Public Health.

[28] Brännlund R. (2008). Productivity and environmental regulations. Umeå Econ. Stud..

[29] Wu J., Xia Q., Li Z. (2022). Green innovation and enterprise green total factor productivity at a micro level: A perspective of technical distance. J. Clean. Prod..

[30] Wang M., Li Y., Liao G. (2021). Research on the impact of green technology innovation on energy total factor productivity, Based on provincial data of China. Front. Environ. Sci..

[31] Song M., Peng L., Shang Y., Zhao X. (2022). Green technology progress and total factor productivity of resource-based enterprises: A perspective of technical compensation of environmental regulation. Technol. Forecast. Soc..

[32] Jiakui C., Abbas J., Najam H., Liu J., Abbas J. (2022). Green technological innovation, green finance, and financial development and their role in green total factor productivity: Empirical insights from China. J. Clean. Prod..

[33] Santra S. (2017). The effect of technological innovation on production-based energy and CO2 emission productivity: Evidence from BRICS countries. Afr. J. Sci. Technol. Innov. Dev..

[34] Meirun T., Mihardjo L.W.W., Haseeb M., Khan S.A.R., Jermsittiparsert K. (2021). The dynamics effect of green technology innovation on economic growth and CO2 emission in Singapore: New evidence from bootstrap ARDL approach. Environ. Sci. Pollut. Res..

[35] Mohd Suki N., Mohd Suki N., Afshan S., Sharif A., Ariff Kasim M., Rosmaini Mohd Hanafi S. (2022). How does green technology innovation affect green growth in ASEAN-6 countries? Evidence from advance panel estimations. Gondwana Res..

[36] Xiao S., He Z., Zhang W., Qin X. (2022). The Agricultural Green Production following the Technological Progress: Evidence from China. Int. J. Environ. Res. Public Health.

[37] Managi S., Opaluch J.J., Jin D., Grigalunas T.A. (2005). Environmental regulations and technological change in the offshore oil and gas industry. Land Econ..

[38] Ambec S., Cohen M.A., Elgie S., Lanoie P. (2013). The porter hypothesis at 20: Can environmental regulation enhance innovation and competitiveness?. Rev. Environ. Econ. Policy.

[39] Guo Q., Zhou M., Liu N., Wang Y. (2019). Spatial Effects of Environmental Regulation and Green Credits on Green Technology Innovation under Low-Carbon Economy Background Conditions. Int. J. Environ. Res. Public Health.

[40] Peng H., Shen N., Ying H., Wang Q. (2021). Can environmental regulation directly promote green innovation behavior?—based on situation of industrial agglomeration. J. Clean. Prod..

[41] Du K., Cheng Y., Yao X. (2021). Environmental regulation, green technology innovation, and industrial structure upgrading: The road to the green transformation of Chinese cities. Energy Econ..

[42] Liu Y., Zhu J., Li E.Y., Meng Z., Song Y. (2020). Environmental regulation, green technological innovation, and eco-efficiency: The case of Yangtze river economic belt in China. Technol. Forecast. Soc. Chang..

[43] Guo Y.Y., Xia X.N., Zhang S., Zhang D.P. (2018). Environmental regulation, government R&D funding and green technology innovation: Evidence from China provincial data. Sustainability.

[44] Xinan L. (2021). Environmental regulation, government subsidies and regional green technology innovation. Econ. Surv..

[45] Behera P., Sethi N. (2022). Nexus between environment regulation, FDI, and green technology innovation in OECD countries. Environ. Sci. Pollut. Res..

[46] Yi M., Fang X., Wen L., Guang F., Zhang Y. (2019). The heterogeneous effects of different environmental policy instruments on green technology innovation. Int. J. Environ. Res. Public Health.

[47] Féres J., Reynaud A. (2012). Assessing the impact of formal and informal regulations on environmental and economic performance of Brazilian manufacturing firms. Environ. Resour. Econ..

[48] Grossman G.M., Krueger A.B. (1991). Environmental Impacts of a North American Free Trade Agreement.

[49] Xin S., Shenshi Z. (2019). Dual environmental regulation, government subsidy and enterprise innovation output. China Popul. Res. Environ..

[50] Li Y., Li F., Li X. (2022). Environmental regulation, digital inclusive finance and urban industrial upgrading—Analysis based on spatial spillover effect and regulation effect. Inq. Econ. Issues.

[51] Liu Y., Zhao X. (2022). Carbon performance, green technology innovation and financial performance —An analysis based on moderating and threshold effects. Chinese Certif. Public Account..

[52] Ren S., Wu H., Ran Q. (2019). Outward foreign direct investment, institutional environment and green total factor productivity—An empirical study based on generalized quantile and dynamic threshold panel model. Int. Bus..

[53] Rusiawan W., Tjiptoherijanto P., Suganda E., Darmajanti L. (2015). Assessment of Green Total Factor Productivity Impact on Sustainable Indonesia Productivity Growth. Procedia Environ. Sci..

[54] Chung Y.H., Färe R., Grosskopf S. (1997). Productivity and undesirable outputs: A directional distance function approach. J. Environ. Manag..

[55] Fukuyama H., Weber W.L. (2009). A directional slacks-based measure of technical inefficiency. Socio-Econ. Plan. Sci..

[56] Jiansheng W., Yan N., Jian P., Zheng W., Xiulan H. (2014). Research on energy consumption dynamic among prefecture-level cities in China based on DMSP/OLS Nighttime Light. Geogr. Res..

[57] Guoqun M., Yanwen T. (2021). Impact of environmental regulation on agricultural green total factor productivity—Analysis based on the panel threshold model. J. Agric. Econ..

[58] Javorcik B.S., Wei S.-J. (2003). Pollution havens and foreign direct investment: Dirty secret or popular myth?. Contrib. Econ. Anal. Policy.

[59] Jingyan F. (2009). An empirical analysis on industrial characteristics, environmental regulation and air pollution: Guangdong’s manufacturing industry. China Popul. Res. Environ..

[60] Li C., Lu X., Li J. (2018). The impact of local government competition pressure on regional production efficiency loss. China Soft Sci..

[61] Hansen B.E. (1999). Threshold effects in non-dynamic panels: Estimation, testing, and inference. J. Econ..

[62] Liu Y., Shao X., Liu S., Ran R. (2022). Driving forces deconstructing green technology innovation dynamics from a spatial perspective: Policy push and market pull. Sci. Technol. Prog. Policy.

[63] Bartik T.J. (1988). The Effects of Environmental Regulation on Business Location in the United States. Growth Chang..

[64] Jorgenson D.W., Wilcoxen P.J. (1990). Environmental Regulation and U.S. Economic Growth. RAND J. Econ..

[65] Xie R., Yuan Y., Huang J. (2017). Different Types of Environmental Regulations and Heterogeneous Influence on “Green” Productivity: Evidence from China. Ecol. Econ..

